# Older Adults' Online and Offline Social Interactions During the COVID-19 Pandemic

**DOI:** 10.1093/geroni/igab046.1167

**Published:** 2021-12-17

**Authors:** Kristina Shiroma, Atami de Main, Nathan Davis, Bo Xie

**Affiliations:** 1 The University of Texas at Austin, The University of Texas at Austin, Texas, United States; 2 School of nursing, the university of texas at Austin, Austin, Texas, United States; 3 The University of Texas at Austin, Austin, Texas, United States

## Abstract

During the COVID-19 pandemic, much of our social interaction has transitioned from in-person to online. This study examined older adults’ social interaction during COVID-19, online and offline. Participants were recruited from community-dwelling older adults in Central Texas. Data collection took place via the telephone during June-August 2020 (N = 200; age range: 65-92 years; Mean: 73.6; SD: 6.33). Participants used a variety of communication modes, including phone or texting (used by 99% of the participants); email (44%); in person (35%); video chat (31%); social media (24%); and postal mail (4%). Most participants (77%) used more than one communication mode. Participants discussed their preferences for and challenges of technology (i.e., smart phones) and its applications (i.e., video chat, telehealth, and social media). Participants’ self-reported experiences ranged from positive (50%), mixed (35%), to negative (15%). These findings will inform policy and community interventions to promote older adults’ social interactions during the pandemic.

